# Supplementary material for: Epidemiological study on foot-and-mouth disease in small ruminants: Sero-prevalence and risk factor assessment in Kenya
Source: PLoS One. 2021 Aug 2;16(8):e0234286. doi: 10.1371/journal.pone.0234286 (PMC8328338; doi:10.1371/journal.pone.0234286)
Supplement: S1 Table — (DOCX) [file pone.0234286.s001.docx]

**S1 Table. FMD sero-prevalence and associated risk factors in SR in various countries**

| **Country** | **Sero-prevalence (%)** | **Associated risk factors** | **Reference** |
| --- | --- | --- | --- |
| Ethiopia | 7.07 in sheep; 7.10 in goats | Agroecology, production system, age | [8] |
| Ethiopia | 4.0-11.0 | Production system, geographic location, species, age, contact with wildlife, season, breed, interaction with other livestock species | [9] |
| India | 23.0 in goats; 12.0 in sheep | Not investigated | [10] |
| Israel | 3.7 | Proximity to herd with outbreak, grazing, herd size | [11] |
| Libya | 13.5 | Not studied | [12] |
| Medina | 27.8 in sheep; 7.9 in goats | Breed | [13] |
| Myanmar | 42.4 | Interaction with infected cattle and pigs, trade area | [14] |
| Nigeria | 41.7 in sheep; 21.8 in goats | Species, geographical location | [15] |
| Pakistan | 21.0 | Age, sex, pregnancy, herd type | [16] |
| Pakistan | 22.8  25.8 in goats; 14.3 in sheep | Species, sex | [17] |
| Sudan | 14.1 | Not studied | [18] |
| Tanzania | 14.1 in 2015; 39.0 in 2014 | Age, sex, species, geographic location, interaction with other herds | [19] |
| Tanzania (northern)  Uganda | 48.5  14 in goats; 22 in sheep | Age, production system, herd size, acquisition of livestock, wildlife interaction  Husbandry practices | [20]  [21] |
