# Supplementary material for: Epidemiological study on foot-and-mouth disease in small ruminants: Sero-prevalence and risk factor assessment in Kenya
Source: PLoS One. 2021 Aug 2;16(8):e0234286. doi: 10.1371/journal.pone.0234286 (PMC8328338; doi:10.1371/journal.pone.0234286)
Supplement: S2 Table — (DOCX) [file pone.0234286.s002.docx]

**S2 Table. Number of sheep and goats in the herds from which animals were sampled in the study area, Kenya, 2016**

| **Pastoral Zone (PZ)** |  |  |  |  |  |  |
| --- | --- | --- | --- | --- | --- | --- |
| **Age category** | **Minimum number of animals in the zone** | **Maximum number of animals in the zone** | **Total number of animals in the zone** | **Mean**  **number of animals in the zone** | **Std. Deviation** | **95% CI of mean** |
| Male<1yr | 1 | 330 | 9,761 | 12.93 | 25.33 | 9.66-14.44 |
| Female<1yr | 1 | 500 | 15,600 | 18.39 | 40.01 | 14.87-22.82 |
| Male≥1yr | 1 | 250 | 12,539 | 14.09 | 23.19 | 12.02-16.60 |
| Female≥1yr | 1 | 750 | 42,696 | 64.68 | 98.94 | 55.53-74.86 |
| Overall Mean | 1 | 458 | 20,149 | 27.50 |  |  |
| **Sedentary zone (SZ)** |  |  |  |  |  |  |
| Male<1yr | 1 | 100 | 1,523 | 1.67 | 5.15 | 1.30-2.20 |
| Female<1yr | 1 | 25 | 1,545 | 1.72 | 2.90 | 1.48-1.98 |
| Male≥1yr | 1 | 300 | 2,409 | 2.82 | 14.51 | 1.90-4.26 |
| Female≥1yr | 1 | 160 | 5,561 | 4.61 | 11.90 | 3.65-5.74 |
| Overall Mean | 1 | 146 | 2,760 | 2.70 |  |  |
