# Supplementary material for: Epidemiological study on foot-and-mouth disease in small ruminants: Sero-prevalence and risk factor assessment in Kenya
Source: PLoS One. 2021 Aug 2;16(8):e0234286. doi: 10.1371/journal.pone.0234286 (PMC8328338; doi:10.1371/journal.pone.0234286)
Supplement: S3 Table — (DOCX) [file pone.0234286.s003.docx]

**S3 Table. Small ruminant FMD Sero-positivity per county, Kenya, 2016**

| **S/No** | **County** | **County type** | **No. of herds** | **Total Tested** | **Negative** | **Positive** | **AP (%)** | **TP (%)** | **95%CI of TP (%)** |
| --- | --- | --- | --- | --- | --- | --- | --- | --- | --- |
| 1 | Mandera | Pastoral | 76 | 313 | 111 | 202 | 64.5 | 64.1 | 58.9-69.8 |
| 2 | Kilifi | Sedentary | 13 | 108 | 55 | 53 | 49.1 | 48.6 | 39.4-58.8 |
| 3 | Lamu | Sedentary | 2 | 28 | 16 | 12 | 42.9 | 42.3 | 25.0-62.6 |
| 4 | Kajiado | Pastoral | 5 | 57 | 35 | 22 | 38.6 | 38.0 | 26.3-52.4 |
| 5 | West Pokot | Pastoral | 42 | 600 | 384 | 216 | 36.0 | 35.4 | 32.1-40.0 |
| 6 | Garissa | Pastoral | 33 | 420 | 275 | 145 | 34.5 | 33.8 | 30.0-39.3 |
| 7 | Turkana | Pastoral | 59 | 515 | 366 | 149 | 28.9 | 28.2 | 25.1-33.1 |
| 8 | Wajir | Pastoral | 64 | 446 | 320 | 126 | 28.3 | 27.6 | 24.2-32.7 |
| 9 | Kwale | Sedentary | 3 | 43 | 31 | 12 | 27.9 | 27.2 | 15.8-43.9 |
| 10 | Tana River | Pastoral | 29 | 375 | 276 | 99 | 26.4 | 25.7 | 22.1-31.2 |
| 11 | Isiolo | Pastoral | 12 | 164 | 123 | 41 | 25.0 | 24.2 | 18.7-32.5 |
| 12 | Marsabit | Pastoral | 37 | 518 | 398 | 120 | 23.2 | 22.4 | 19.7-27.1 |
| 13 | Samburu | Pastoral | 24 | 343 | 264 | 79 | 23.0 | 22.2 | 18.6-27.9 |
| 14 | Nyeri | Sedentary | 15 | 222 | 174 | 48 | 21.6 | 20.8 | 16.5-27.7 |
| 15 | Uasin-Gishu | Sedentary | 6 | 57 | 45 | 12 | 21.1 | 20.3 | 11.8-34.2 |
| 16 | Makueni | Sedentary | 20 | 189 | 146 | 43 | 22.8 | 22.0 | 17.1-29.5 |
| 17 | Machakos | Sedentary | 14 | 210 | 170 | 40 | 19.0 | 18.2 | 14.1-25.2 |
| 18 | Kericho | Sedentary | 7 | 98 | 80 | 18 | 18.4 | 17.6 | 11.5-27.7 |
| 19 | Tharaka-Nithi | Sedentary | 3 | 99 | 82 | 17 | 17.2 | 16.4 | 10.6-26.4 |
| 20 | Siaya | Sedentary | 23 | 101 | 85 | 16 | 15.8 | 14.9 | 9.6-24.8 |
| 21 | Narok | Pastoral | 11 | 131 | 110 | 21 | 16.0 | 15.2 | 10.4-23.7 |
| 22 | Kitui | Sedentary | 17 | 181 | 152 | 29 | 16.0 | 15.2 | 11.2-22.4 |
| 23 | Nandi | Sedentary | 23 | 198 | 168 | 30 | 15.2 | 14.3 | 10.6-21.1 |
| 24 | Kisumu | Sedentary | 51 | 182 | 155 | 27 | 14.8 | 13.9 | 10.2-21.0 |
| 25 | Nyandarua | Sedentary | 4 | 55 | 47 | 8 | 14.5 | 13.6 | 6.9-27.2 |
| 26 | Homa Bay | Sedentary | 36 | 146 | 126 | 20 | 13.7 | 12.8 | 8.8-20.6 |
| 27 | Bomet | Sedentary | 10 | 140 | 121 | 19 | 13.6 | 12.7 | 8.6-20.6 |
| 28 | Meru | Sedentary | 29 | 209 | 181 | 28 | 13.4 | 12.5 | 9.2-19.0 |
| 29 | Baringo | Sedentary | 39 | 241 | 210 | 31 | 12.9 | 12.0 | 9.0-17.9 |
| 30 | Taita/Taveta | Sedentary | 3 | 42 | 37 | 5 | 11.9 | 11.0 | 4.5-26.4 |
| 31 | Laikipia | Sedentary | 4 | 56 | 50 | 6 | 10.7 | 9.8 | 4.4-22.6 |
| 32 | Nakuru | Sedentary | 11 | 76 | 68 | 8 | 10.5 | 9.6 | 5.0-20.2 |
| 33 | Kakamega | Sedentary | 23 | 132 | 119 | 13 | 9.8 | 8.9 | 5.6-16.6 |
| 34 | Kiambu | Sedentary | 11 | 85 | 78 | 7 | 8.2 | 7.3 | 3.4-16.8 |
| 35 | Bungoma | Sedentary | 17 | 93 | 86 | 7 | 7.5 | 6.6 | 3.3-15.4 |
| 36 | Embu | Sedentary | 6 | 81 | 75 | 6 | 7.4 | 6.5 | 3.1-16.0 |
| 37 | Kisii | Sedentary | 18 | 70 | 65 | 5 | 7.1 | 6.2 | 2.7-16.6 |
| 38 | Elgeiyo-Marakwet | Sedentary | 20 | 168 | 158 | 10 | 6.0 | 5.1 | 3.1-11.0 |
| 39 | Busia | Sedentary | 20 | 142 | 134 | 8 | 5.6 | 4.6 | 2.6-11.2 |
| 40 | Kirinyaga | Sedentary | 4 | 59 | 57 | 2 | 3.4 | 2.4 | 0.6-12.8 |
| 41 | Vihiga | Sedentary | 14 | 58 | 57 | 1 | 1.7 | 0.7 | 0.1-10.5 |
| 42 | Muranga | Sedentary | 8 | 94 | 93 | 1 | 1.1 | 0.1 | 0.1-6.6 |
| 43 | Mombasa | Sedentary | 1 | 5 | 5 | 0 | 0.0 | -1.0 | 0.0-53.7 |
| 44 | Nyamira | Sedentary | 5 | 14 | 14 | 0 | 0.0 | -1.0 | 0.0-26.8 |
|  | **Overall** | **All** | **872** | **7564** | **5802** | **1762** | **23.3** | 22.5 | **22.3-24.3** |

AP is apparent prevalence; TP is true prevalence. Calculation of the CIs of TP uses the formula

$p\pm1.96\surd(\frac{\mathrm{pq}}{\mathrm{nJ}2})$.This is equivalent to that of simple proportion p±1.96√pq/n in near perfect tests as in our study as J (Youden’s index)=(Se+Sp-1) nears 1. Thus CIs of TP are similar to those of AP.
