# Supplementary material for: Epidemiological study on foot-and-mouth disease in small ruminants: Sero-prevalence and risk factor assessment in Kenya
Source: PLoS One. 2021 Aug 2;16(8):e0234286. doi: 10.1371/journal.pone.0234286 (PMC8328338; doi:10.1371/journal.pone.0234286)
Supplement: S1 File — (DOCX) [file pone.0234286.s004.docx]

**S1 File *DATA COLLECTION QUESTIONNAIRE_CROSSECTIONAL SURVEY_CBPP, FMD, PPR, RVF – PAGE 1***

Serial No..................... Enumerator Name................................................................................

**A. GENERAL INFORMATION**

| Herd number |  |  |  | Name of livestock keeper *(2or 3 Names)* and Mobile Tel. No. |  |
| --- | --- | --- | --- | --- | --- |
| Date |  | | | Name of village |  |
| GPS  coordinates | Lat: | | | Sub-Location |  |
|  | Long: | | |  |  |
| Elevation |  | | | Location |  |
| Distance to  main road | Km | | |  |  |

B: **PRODUCTION SYSTEM**

1. Herd size: *how many animals of different species and ages are in the herd?*

| Species | Young  (<1 year sheep, goats, pigs. <2 years cattle and camels) | | Mature  (1 year or more - sheep, goats, pigs. 2 years or more - cattle and camels) | | Total |
| --- | --- | --- | --- | --- | --- |
|  | Male | Female | Male | Female |  |
| Cattle |  |  |  |  |  |
| Sheep |  |  |  |  |  |
| Goats |  |  |  |  |  |
| Pigs |  |  |  |  |  |
| Camels |  |  |  |  |  |

2. Herd dynamics*: in the past year, how many animals have been added to the herd - births, purchases, gifts etc.? In the past year, how many animals have left the herd - death, sale, slaughter, gifts etc.?*

| Species | No.  born | No.  died | No.  bought | No.  sold | No.  slaughtered | No.  gifted or loaned | No. gifts  received or borrowed | Other reasons  for leaving herd (stolen, lost, predator) | Other reasons  for joining herd (stray animal, breeding bull) |
| --- | --- | --- | --- | --- | --- | --- | --- | --- | --- |
| Cattle |  |  |  |  |  |  |  |  |  |
| Sheep |  |  |  |  |  |  |  |  |  |
| Goats |  |  |  |  |  |  |  |  |  |

| Species | 3. Production type: *what is*  *the main reason for keeping each species?* | | | 4. Production system: *what is the main*  *farming system?* | | | 5. Housing: *are the animals*  *enclosed?* | |
| --- | --- | --- | --- | --- | --- | --- | --- | --- |
|  | Dairy | Meat | Multi-  purpose | Sedentary/  mixed farming | Agro-  pastoralist | Pastoralist | Enclosed at  night | Enclosed  during day |
| Cattle |  |  |  |  |  |  |  |  |
| Sheep |  |  |  |  |  |  |  |  |
| Goats |  |  |  |  |  |  |  |  |

| Species | 6. Grazing: *what type of grazing is practised?* | | | | 7. Water source: *where do the animals drink?* | |
| --- | --- | --- | --- | --- | --- | --- |
|  | Zero-grazed | Fenced | Communal | Migratory | On the farm | Shared water source |
| Cattle |  |  |  |  |  |  |
| Sheep |  |  |  |  |  |  |
| Goats |  |  |  |  |  |  |
|  |  |  |  |  |  |  |

***Data Collection Questionnaire_CrosSectionalSurvey_CBPP, FMD, PPR, RVF*** *–* ***P*age 2**

| Herd no. |  |  |  | Date |  | Name of livestock keeper |  | Name of village/location |  |
| --- | --- | --- | --- | --- | --- | --- | --- | --- | --- |

| Species | 8. Breeding method | | | |
| --- | --- | --- | --- | --- |
|  | AI | Own bull ram or he -goat | Own bull /ram or he goat common use | bull /ram or he goat from another farm |
| Cattle |  |  |  |  |
| Sheep |  |  |  |  |
| Goats |  |  |  |  |

9. Selling live animals: *in the past one year, what methods have you used to sell animals?*

| Species | Livestock market | Direct local sale to neighbour  or nearby village | Direct sale to middle  man/trader | Other (specify) |
| --- | --- | --- | --- | --- |
| Cattle |  |  |  |  |
| Sheep |  |  |  |  |
| Goats |  |  |  |  |

10. Buying live animals: *in the past one year, what methods have you used to buy animals?*

| Species | Livestock market | Direct purchase from  neighbour or nearby village | Direct purchase from  middle man/trader | Other |
| --- | --- | --- | --- | --- |
| Cattle |  |  |  |  |
| Sheep |  |  |  |  |
| Goats |  |  |  |  |

11. Wildlife –Livestock Interaction

| Do your animals come in close contact with wildlife at any time*(Tick)* | If yes which type of wild animals*(Name them)* | Interaction points:  1=Grazing grounds  2=Watering points  3= others(specify) |
| --- | --- | --- |
|  |  |  |

12. Personnel, equipment and farm products movement at farm level

| Do you share Equipment |  | Milk collection in farm |  | Buy or sell hay  1=buy 2=sell |  |
| --- | --- | --- | --- | --- | --- |
| Do you Share Laborers with neighbors |  | AI Attendant/ Vet visit |  | Buy or sell compost: 1=buy 2=sell |  |

***Data Collection Questionnaire_CrosSectionalSurvey_CBPP, FMD, PPR, RVF*** *–* ***P*age 3**

| Herd no. |  |  |  | Date |  | Name of livestock keeper |  | Name of village/location |  |
| --- | --- | --- | --- | --- | --- | --- | --- | --- | --- |

C: DISEASE SITUATION

13. *What diseases have affected the herd in the past one year?*

| Local disease  name | Species  affected | Number  affected | Main clinical signs | No.  affected | No. Dead | Duration of disease | Season(s) when  occurred | Suspected  diagnosis |
| --- | --- | --- | --- | --- | --- | --- | --- | --- |
|  |  |  |  |  |  |  |  |  |
|  |  |  |  |  |  |  |  |  |
|  |  |  |  |  |  |  |  |  |
|  |  |  |  |  |  |  |  |  |
|  |  |  |  |  |  |  |  |  |
|  |  |  |  |  |  |  |  |  |
|  |  |  |  |  |  |  |  |  |

**14. Rank the 5 most important cattle diseases/conditions and give the criteria for ranking (*1 is most important and 5 is least important)***

| Rank | Disease | Criterion |
| --- | --- | --- |
| 1 |  |  |
| 2 |  |  |
| 3 |  |  |
| 4 |  |  |
| 5 |  |  |

***Data Collection Questionnaire_CrosSectionalSurvey_CBPP, FMD, PPR, RVF*** *–* ***P*age 4**

| Herd no. |  |  |  | Date |  | Name of livestock keeper |  | Name of village/location |  |
| --- | --- | --- | --- | --- | --- | --- | --- | --- | --- |

15. Current diseases affecting the herd: (using the local terms for FMD-, CBPP-, PPR- and RVF-like disease syndromes) *ask if there are any animals in the herd that are currently sick with any of these syndromes?*

If there is no local name for any of these diseases, describe the clinical signs of the disease and ask if they have seen anything like this in the last one year (fill details above), and if they currently have any sick animals that might have any of these diseases (fill details below).

| Local disease  name | Species  affected | Number  affected | Main clinical signs | Season(s) when  occurred | Suspected  diagnosis |
| --- | --- | --- | --- | --- | --- |
|  |  |  |  |  |  |
|  |  |  |  |  |  |
|  |  |  |  |  |  |
|  |  |  |  |  |  |
|  |  |  |  |  |  |
|  |  |  |  |  |  |

Ask the owner to show you the clinical cases after you have finished this interview, and fill in the clinical examination form.

16. *If an animal becomes sick, what do you usually do? (tick all that apply)*

| Do  nothing | Treat it  myself (or a family member) | Consult a  traditional healer | Consult a  community animal health worker | Consult an  extension officer | Consult a  private vet | Consult a  government vet | Consult an  NGO | Others (specify) |
| --- | --- | --- | --- | --- | --- | --- | --- | --- |
|  |  |  |  |  |  |  |  |  |

Reasons for choice above...................................................................................................................................................

***Data Collection Questionnaire_CrosSectionalSurvey_CBPP, FMD, PPR, RVF*** *–* ***Page* 5**

| Herd no. |  |  |  | Date |  | Name of livestock keeper |  | Name of village/location |  |
| --- | --- | --- | --- | --- | --- | --- | --- | --- | --- |

17. *Where do you get medicines from?* (tick all that apply)

| Collect or  make myself | Traditional  healer | Community  animal health worker | Pharmacy/  Agro-vet | General  shop or market stall | Private vet | Government  vet | NGO | Others (specify) |
| --- | --- | --- | --- | --- | --- | --- | --- | --- |
|  |  |  |  |  |  |  |  |  |

20. *When did you last have vaccinations against the following diseases?*

| Species | Vaccine | Date of vaccination | Source of vaccine | *Reasons for vaccinating |
| --- | --- | --- | --- | --- |
|  | FMD |  |  |  |
|  | PPR |  |  |  |
|  | RVF |  |  |  |
|  | CBPP |  |  |  |

*Reasons for vaccinating: 1=Routine 2=Rumor of disease nearby 3=Outbreak within herd 4=Ring vaccination 5=others (specify)

Use of insecticides or acaricides:

| Species | 21. *Do you use tick or*  *fly control?* | | 22. *If yes, what method do you use?* | | | | 23. *If you dip, is it private or*  *Communal?* | |
| --- | --- | --- | --- | --- | --- | --- | --- | --- |
|  | Yes | No | Hand  dressing | Spray | Dip | Others (specify) | Private | Communal |
| Cattle |  |  |  |  |  |  |  |  |
| Sheep |  |  |  |  |  |  |  |  |
| Goats |  |  |  |  |  |  |  |  |

**AU-IBAR – STSD Project and VS of Kenya**

**Guidelines for Filling in the Survey Questionnaire**

**Herd number:** insert unique herd number for each herd from which samples are collected.

**Date:** insert the date on which samples were collected using day/month/year format e.g. 25/6/2014.

**GPS Coordinates:** insert the latitude and longitude from a GPS device.

**Elevation:** insert the elevation (in metre above sea level).

**Distance to main road:** ask the owner how far it is to the nearest main road, estimated in km. A main road could be surfaced or unsurfaced, but runs from a city, town or major village to another city, town or major village.

**Name of livestock keeper:** insert the name of the livestock owner.

**Name of village/location:** insert the name of the village in which the herd is located

**Admin area 1:** insert the name of the lowest administrative area to identify the location of the village. This will depend on terminology in each country.

**Admin area 2 and higher admin divisions:** insert the name of the next higher levels of administrative divisions to identify the location of the village. This will depend on terminology in each country and number of higher level administrative divisions.

1. **Herd size:** ask the owner what species of animals they are keeping, and approximately how many males and females, young and old of each species. For cattle and camels, young animals are defined as those aged less than 2 years of age. For sheep, goats and pigs, young animals are defined as those aged less than 1 year of age.

2. **Herd dynamics:** for each species, ask the owner how many animals have been added to the herd in different ways during the last one year, this means – how many animals were born, how many animals did they buy, how many animals did they receive as gifts or on loan? And how many animals joined the herd for any other reason e.g. a bull brought in for breeding, a stray animal that was found? Then ask how many animals have left the herd for different reasons during the past one year, this means – how many animals died, how many were sold, how many were slaughtered, how many where given away as gifts or loans, how many animals left the herd for other reasons e.g. killed by a predator, stolen, lost etc.?

3. **Production type:** for each species, ask what is the main purpose of keeping these animals? Is it for milkproduction (dairy), meat-production (meat) or for multiple uses e.g. milk, meat, draught etc. Tick the relevant box.

4. **Production system:** what is the main production system being used by the livestock owner? We have provided three broad categories – tick the one that is most relevant:  sedentary mixed farming is where the farm combines crop production and livestock production, and the herd remains in one place throughout the year, although it may move away from the farm for grazing or water on a daily basis.  agro-pastoralist is where the livestock owner may do some crop production as well as livestock keeping, but livestock-keeping is a major activity, and in most years the herd moves away from the home location for part of the year in search of water or grazing.  pastoralist is where only livestock are kept, and in most years the herd moves away from the home location for part of the year in search of water or grazing.

Page – 1

5. **Housing:** is the herd housed or enclosure during the night or during the day? This may be inside a building or in a fenced enclosure. If they are housed or enclosed all the time (zero-grazed) then tick both night and day boxes.

6. **Grazing:** for each species, what type of grazing system is practised?  zero-grazed - all fodder is cut and carried to the animals, they do not leave the farm.  fenced – the animals graze or browse within an enclosed area on the farm, they do not leave the farm.  communal – the animals graze or browse in an area that is shared with herds belonging to other people, but they return to the farm each night.  migratory – the animals move to different locations for some or all of the year to graze and browse, and this is shared with herds belonging to other people.

7. **Water source:** for each species where do they get their water?  on the farm – the animals have a water source on the farm that is not shared with other herds.  shared water source – the animals’ water source is shared with other herds.

8. **Selling live animals:** for each species, during the past one year, how have they sold live animals? They may have used more than one method so tick all the relevant boxes.  livestock market – the animals were taken to a livestock market for sale  direct local sale – the animals were sold directly to a neighbour or to someone in a nearby place, without going to a market  direct sale to middleman/trader – the animals were sold directly to a trader or middleman, without going to a market (although the trader might subsequently have taken them to a market)  other – if they have sold animals by another method, give a description.

9. **Buying live animals:** for each species, during the past one year, how have they purchased live animals? They may have used more than one method so tick all the relevant boxes.  livestock market – the livestock keeper went to a market to buy animals.  direct purchase – the livestock keeper bought animals directly from a neighbour or from a nearby village, without going to a market  direct purchase from middleman/trader – the livestock keeper bought animals directly from a trader or middleman, without going to a market  other – if they have bought animals by any other method, give a description

Page – 2

Fill in the herd number, date, name of livestock keeper and name of village/location as on page 1 – this is to ensure that the questionnaire can be identified if for some reason the pages become separated from page 1.

10. **What diseases have affected the herd in the past one year:** ask the livestock keeper what diseases have affected each species in the past one year. For each disease that is mentioned, ask the livestock keeper and write down:

 the local name that is used for the disease  the different species that are affected  the number of animals of each species that were affected by the disease during the past one year  a description of the main clinical signs that the livestock keeper has seen  during which season or seasons does the disease occur, or can it happen at any time in the year?  based on the description of the disease, write down the most likely disease diagnosis.

Take particular notice of any disease syndromes that are described that could be one of the four diseases of interest – FMD, CBPP, PPR and RVF – and use the local names for these syndromes in the following question.

11. **Current diseases affecting the herd:** ask the livestock keeper whether he or she has any animals at the moment that are sick with FMD, CBPP, PPR or RVF. Use the local disease terms that the livestock keeper used in question 10, or local terms that are in common use in the area. If there is no local term for any of the four diseases, then give a short description of the main clinical signs, and ask if they have any animals with these signs at the moment.

For each of the four diseases, ask the livestock keeper and write down:

 the local disease name  the species that are currently affected  the number of animals that are currently affected in each species  the main clinical signs that they are showing  the current season  the most likely disease diagnosis.

Once you have completed the questionnaire, ask the livestock keeper to show the clinical cases that have been described to you, and use the clinical examination form to record your findings.

Page – 3

Fill in the herd number, date, name of livestock and keeper and name of village/location as on page 1 – this is to ensure that the questionnaire can be identified, if for some reason the pages become separated from page 1.

12. **If an animal becomes sick, what do you usually do**? Ask the owner what they normally do when one or more of their animals becomes sick. They are likely to take different actions for different types of disease and species of animal, so tick all the boxes that are relevant.

13. **Where do you get medicines from?** Ask the owner where they normally get medicines from, whether local traditional medicines or modern medicines. Again this may depend on the type of disease and species of animal, so tick all the boxes that are relevant.

14. **When did you last have vaccinations against the following diseases?** For each of the four diseases, FMD, PPR, RVF and CBPP, ask when the herd was last vaccinated. For each vaccine, record all the species that were vaccinated, the date of vaccination (this could be the month and year, or just the year if the owner can’t remember the month), and the source of the vaccine e.g. government veterinary office, private pharmacy, NGO, etc.

15. **Use of insecticides or acaricides**. Ask the owner for each species – do you use tick or fly control? Put a tick in the relevant box for each species – yes or no. If the owner answers no, then there is no need to ask questions 16 and 17.

16**. If the owner says they do use tick or fly control, then for each species ask what method do they use?** Put a tick in the relevant boxes.

17. From question 16, if the owner doesn’t use a dip, then there is no need to ask question 17. If the owner does use a dip, then ask if it is private (used only by the owner’s herd) or communal (used by other herds) and tick the relevant box.

AU-IBAR – STSD and VS of Kenya

***Species------------------------- STSD Survey Sample Collection Form***

| **Date of Sampling** |  |  | **Herd number** | |  | |  | |  | | |  | | **Name of Owner** |  | | **Name of village** |  | **Admin area** |  | | | |
| --- | --- | --- | --- | --- | --- | --- | --- | --- | --- | --- | --- | --- | --- | --- | --- | --- | --- | --- | --- | --- | --- | --- | --- |
| Sample No. | Breed | Species | Sex  (tick box) | | Dentition (Pairs permanent incisors (tick box) | | | | | | | | | Origin  (Born in herd –tick box, Brought in – date & source) | | | Clinical signs  (describe) | | | Vaccinations  (date of vaccination / N=no / U=unknown) | | | |
|  |  |  | M | F | 0 | 1 | | 2 | | 3 | 4 | | 4* | Born in herd | | Brought in |  |  |  | CBPP | FMD | RVF | PPR |
|  |  |  |  |  |  |  | |  | |  |  | |  |  | |  |  | | |  |  |  |  |
|  |  |  |  |  |  |  | |  | |  |  | |  |  | |  |  | | |  |  |  |  |
|  |  |  |  |  |  |  | |  | |  |  | |  |  | |  |  | | |  |  |  |  |
|  |  |  |  |  |  |  | |  | |  |  | |  |  | |  |  | | |  |  |  |  |
|  |  |  |  |  |  |  | |  | |  |  | |  |  | |  |  | | |  |  |  |  |
|  |  |  |  |  |  |  | |  | |  |  | |  |  | |  |  | | |  |  |  |  |
|  |  |  |  |  |  |  | |  | |  |  | |  |  | |  |  | | |  |  |  |  |
|  |  |  |  |  |  |  | |  | |  |  | |  |  | |  |  | | |  |  |  |  |
|  |  |  |  |  |  |  | |  | |  |  | |  |  | |  |  | | |  |  |  |  |
|  |  |  |  |  |  |  | |  | |  |  | |  |  | |  |  | | |  |  |  |  |
|  |  |  |  |  |  |  | |  | |  |  | |  |  | |  |  | | |  |  |  |  |
|  |  |  |  |  |  |  | |  | |  |  | |  |  | |  |  | | |  |  |  |  |
|  |  |  |  |  |  |  | |  | |  |  | |  |  | |  |  | | |  |  |  |  |
|  |  |  |  |  |  |  | |  | |  |  | |  |  | |  |  | | |  |  |  |  |
|  |  |  |  |  |  |  | |  | |  |  | |  |  | |  |  | | |  |  |  |  |

**FMD Survey data codebook**

| **Variable** | **0** | **1** | **2** | **3** | **4** | **5** |
| --- | --- | --- | --- | --- | --- | --- |
| **County type** | Pastoralist | Sedentary | - | - | - | **-** |
| **Result** | Negative | Positive | Unidentified | - | - | **-** |
| **Species** | Caprine | Ovine | Unidentified | - | - | **-** |
| **Breed** | Local | Cross | Exotic | Unidentified | - | **-** |
| **Sex** | Female | Male | Unidentified | - | - | **-** |
| **Age** | Mature | Young | Unidentified | - | - | **-** |
| **Origin** | Born-in-herd | Brought-in | Unidentified | - | - | **-** |
| **Production zone** | Pastoral | Sedentary | - | - | - | **-** |
| **Brought in SR** | No | Yes | Unidentified | - | - | **-** |
| **Buy SR from market/middlemen** | No | Yes | - | - | - | **-** |
| **Wildlife interaction** | Yes | No | Unknown | - | - | **-** |
| **Production type** | Meat | Multipurpose | Mixed | Dairy | Unidentified | **-** |
| **Production system** | Sedentary/mixed | Pastoral | Agropastoral | Multiple | Unidentified |  |
| **Housing** | Enclosed at night | None | Enclosed day and night | - | - | **-** |
| **Grazing** | Communal | Fenced | Mixed | Migratory | Unidentified | **Zero** |
| **Watering** | Shared | On-farm | Unidentified | Mixed |  |  |
| **Breeding method** | Own-male | mixed | Common-use-male | Unidentified | Male-from-another-farm | **AI** |
| **Elevation** | ≤1500m above sea level | >1500m above sea level | - | - | - | **-** |
