# Supplementary material for: Epidemiological study on foot-and-mouth disease in small ruminants: Sero-prevalence and risk factor assessment in Kenya
Source: PLoS One. 2021 Aug 2;16(8):e0234286. doi: 10.1371/journal.pone.0234286 (PMC8328338; doi:10.1371/journal.pone.0234286)
Supplement: Attachment — Submitted filename: Response to Reviewers.docx [file pone.0234286.s005.docx]

Salome Kairu-Wanyoike,

Meat Training Institute,

Directorate of Veterinary Services,

State Department of Livestock,

P.O. Box 55-00204,

Athi-River, Kenya.

February 5 2021

The Editor

PLoS ONE Journal

Dear Editor,

I write to submit the corrected manuscript “Epidemiological study on foot-and-mouth disease in small ruminants: sero-prevalence and risk factor assessment in Kenya” as a full research article to *PLoS ONE.*

All the authors reviewed this corrected manuscript and agree to the submission. There are no opposed reviewers. We very much appreciate the opportunity that has been offered to us to correct the manuscript and the valuable inputs by the reviewers. Appended is the response to reviewers on issues raised.

Sincerely,

Salome Kairu-Wanyoike, Ph.D.

 2. In your Methods section, please provide additional location information of the study sites, including geographic coordinates for the data set if available.

 Provided in an updated file now S2 file

3. We note that Figure1, 2, 3 in your submission containmap images which may be copyrighted. All PLOS content is published under the Creative Commons Attribution License (CC BY 4.0), which means that the manuscript, images, and Supporting Information files will be freely available online, and any third party is permitted to access, download, copy, distribute, and use these materials in any way, even commercially, with proper attribution. For these reasons, we cannot publish previously copyrighted maps or satellite images created using proprietary data, such as Google software (Google Maps, Street View, and Earth). For more information, see our copyright guidelines: <http://journals.plos.org/plosone/s/licenses-and-copyright>.

**Figures 2 and 3 were produced as part of the project work by part of the authors of this manuscript who were the principal investigators in the project. The figures may however be in project reports and documents which may appear online elsewhere. Figures 1 and 3 have been removed. Figure 2 has been retained to give a pictorial overview of the study zones and sampling sites.**

[Note: HTML markup is below. Please do not edit.]

Reviewers' comments:

Reviewer's Responses to Questions

**Comments to the Author**

1. Is the manuscript technically sound, and do the data support the conclusions?

The manuscript must describe a technically sound piece of scientific research with data that supports the conclusions. Experiments must have been conducted rigorously, with appropriate controls, replication, and sample sizes. The conclusions must be drawn appropriately based on the data presented.

Reviewer #1: Partly

**This has been addressed.**

Reviewer #2: Yes

2. Has the statistical analysis been performed appropriately and rigorously?

Reviewer #1: No

**This has been addressed.**

Reviewer #2: Yes

3. Have the authors made all data underlying the findings in their manuscript fully available?

The [PLOS Data policy](http://www.plosone.org/static/policies.action#sharing) requires authors to make all data underlying the findings described in their manuscript fully available without restriction, with rare exception (please refer to the Data Availability Statement in the manuscript PDF file). The data should be provided as part of the manuscript or its supporting information, or deposited to a public repository. For example, in addition to summary statistics, the data points behind means, medians and variance measures should be available. If there are restrictions on publicly sharing data—e.g. participant privacy or use of data from a third party—those must be specified.

Reviewer #1: Yes

Reviewer #2: Yes

4. Is the manuscript presented in an intelligible fashion and written in standard English?

PLOS ONE does not copyedit accepted manuscripts, so the language in submitted articles must be clear, correct, and unambiguous. Any typographical or grammatical errors should be corrected at revision, so please note any specific errors here.

Reviewer #1: Yes

Reviewer #2: Yes

5. Review Comments to the Author

Please use the space provided to explain your answers to the questions above. You may also include additional comments for the author, including concerns about dual publication, research ethics, or publication ethics. (Please upload your review as an attachment if it exceeds 20,000 characters)

Reviewer #1: Comments of the authors
General comments
The study on ‘Epidemiological study on foot-and-mouth disease in small ruminants: sero-prevalence and risk factor assessment in Kenya’ is a good and useful study that documented the prevalence of the disease at national level and identified risk factors of the disease that can assist risk based surveillance and control intervention in Kenya. The study has collected quite large and fairly representative sample of the country’s small ruminant population that could generate reliable results.

**Compliments appreciated**

However the manuscript needs improvement in several ways and the following comments are forwarded to improve the manuscript
The introduction is very long. Eight page introduction for research article is unusual.

**The introduction has been reduced considerably.**
There is lack of clarity in in the sampling procedure and sample size determination. The sampling procedure is not clear. Specially the term ‘herd’ was defined in different ways and used inconsistently. This made difficult to understand how the multistage sampling method was used. It was not also clear why the sample size was determined independently for the two zones (PZ and SZ). The same inputs (parameters) were used in each zone and the same number of herds and animals were taken from each zone. If the interest was to stratify the sample, the nationally determined sample size could have been divided among the zones.

**These have been clarified in the various sections.**
The risk factor analysis was done using chi-square, and bivariable (I would suggest to name this univariable) and multivariable logistic regressions. If univariable logistic regression is done, the chi-square analysis is redundant and anything about chi-square in the manuscript should be removed. Looking in to that analysis even the univariable logistic regression is not important as it has not been used for screening the potential risk factor for the multivariable analysis. For that matter, given the adequate sample size, screening the variables is not needed and the univariable analysis can be ignored.

**Chi-square analysis and univariable regression analysis for test of association have been removed. The only Chi-square analyses remaining in the manuscript are for the test of difference in proportions.**

Another problem with the analysis was; while the sampling procedure is cluster sampling, the analysis did not consider the sampling procedure. If cluster sampling is used there will underestimation of standard error (unwarranted significant p-values for regression coefficients) and this has to be taken care of. I would suggest use of mixed effect logistic regression with ‘herds’ and ‘villages’ as random effect variables for more reliable identification of factors associated with the disease.

**Generalized linear mixed effects logistic regression models have been run with county and villages as random effect variables. Interactions between fixed effect variables has also been tested.**
The result has unnecessary detail and redundant results. In line with the comments given in statistical analysis above, the chi-square test and the univariable analysis provides the same result and there is no any need to do the chi-square analysis. The univaraible logistic regression give more information like crude odds ratio for each category of the categorical variables which is not directly possible in the chi-square analysis. So I suggest anything related the chi-square analysis. Even the importance of the univariable analysis result should be re-considered.

**Removed as stated above.**
The discussion of the risk factor should be based on the significance of risk factors in the final multivariable model not on crude association seen from the univariable analysis.

**Chi-square and univariable analyses have been removed**

All the conclusions should be supported by the study findings which was not the case for some the recommendations

**This has been done**
The manuscript also needs improvement in the English.

**English has been corrected throughout the manuscript
Specific Comments
Abstract**
The abstract followed unstructured format. In this type format the abstract should be written in one paragraph.

**The abstract is now in one paragraph**

Line 33-32. No need of mentioning the statistical software in the abstract.

**Statistical software has been removed on Line 33/34**

Line 34-5. If logistic regression is done, the use chi-square for risk factor analysis will be redundancy

**Chi-square has been removed in Line 36**

Line 39 – 40 . Re -write it as “ the risk factors that were significantly positively associated with FMD sero-positivity in SR were being multipurpose (OR=1.150; p=0.034) and dairy (OR=2.029; p=0.003) production types.”

**Done in Line 39-41**

Line 51…’carrier SR’…... Subclinical carrier? You didn’t have any result that showed SR are acting as carriers FMD.

**The term ‘carrier’ has been removed on Line 57**

**Introduction**
The introduction is very long it has to be shortened to maximum of not more than 2-3 pages. E.g. the extensive discussion about differential diagnosis and the different diagnostic assays can be removed. The extensive description of the small ruminant production system in Kenya can be shorted and taken to material and methods part. The extensive discussion about the seroprevalence of SR FMD and production systems worldwide can be shortened or removed.

**The introduction has been shortened.**

Line 154. They ARE……

**Corrected on Line 67.**
Line 166. Average HERD SIZE of sheep and goats

**Corrected on Line 385 in materials and methods.**
Line 176. .
Line 183. Is FMD important in sheep and goat as cause of production loss or for its epidemiological contributions for cattle? Just compare this with next paragraph (line 186-19)

**Sentence has been removed.**
Lin 2011-15. Do these studies support your claim that trade costs are more important than direct cost of FMD for households? Do these SM keeper households participate international trade to countries that free of the disease?

**Sentence has been removed**
Line 226-230. The two sentences seem contradictory

**The two sentences have been removed.**
Materials and methods
Line 248-9. ……………...since FMD is a transboundary disease and also transmitted through export of animals and animal products.

**Corrected on Line 145-147**
Line 255-57. ‘The study targeted the sub location’ this is not clear. In epidemiology target population has its own technical meaning. The target population in this study is the national SR population. Rephrase this sentence to write what you mean.

**Rephrased on Line 341-342**
Line 252-7. Although the section title is study area, the text in this paragraph is more about study population

**A study population section has been created**
Line 283. Sample size calculation was in two stages and per zone) IN EACH STRATUM:

**Included in Line 394**
Line 286. Make clear what is in-contact farm

**Clarified on Line 397-398**
Line 287 …………… “assuming a simple random sample of herds in each stratum independently” not clear

**The phrase has been deleted in Line 398-399**
Line 294 …………’simple random sample” SIMPLE RANDOM SAMPLING? But two line down it describes a cluster sampling in which first sub-locations are selected then household herds and then animals?

**Phrase has been removed in Line 405-406.**
Line 297. ‘323 sub-locations’. The 323 were herds which were defined as farms/in contact farms not sub-location! Again on next line it says “one village (herd)” and ‘”household herd per village”. Please make clear what herd is and use it consistently; not “herd” one time and “household herd’ another time unless they are meant different things.

**Clarified on Line 408-413. But note that because it was one village per sub-location and one herd per village, it is appropriate to use the terms interchangeably but one term has now been maintained for consistency.**
If the interest is to do stratified sampling the approach could have been determining the sample size using one of the sampling techniques (looks cluster sampling in this study case) and allocating the sample among the strata proportionally or if there reason not to allocate proportionally use other method of allocation. I couldn’t see the need to determine the sample size for the two strata independently as all the parameter used are the same for each stratum.

**The cluster sampling was more appropriate given the administrative structure of the country. The two zones are quite distinct in structure even if FMD dynamics (in cattle) seem not very different. The SZ has small counties with very many sub-locations while the PZ has large counties with fewer sub-locations which could have led to high sampling in the SZ and low sampling in the PZ. It was also important to see the difference in the two zones and we chose to have a complete separation between the two and sample equal number of sub-locations in each. This is also explained in line 414-421.**
Line 243. What are field freezers?

**Clarified on Line 461**
Line 343 -344 . ‘One aliquot was used to test for the presence or absence of antibodies of Rift Valley Fever (RVF) and Peste de Petits Ruminants (PPR) antibodies according to the objective of the STSD project’ Give some explanation about this work and make clear that the present FMD work is a part or accompany of that work.

**Explained in Line 465-468**

Line 363-63. Rewrite the sentence avoiding repetitions and put the right reference for the test kit (product, Manufacture Company and place/country).

**Done in Line 488-492**
Line 365-67 On the seropositivity/negativity to FMDV antibodies the outcome variables were categorised based on the on the results of the 3ABC blocking enzyme-linked immunosorbent assay.

**Corrected in Line 492-495**
Line 367-73. The test procedure is not clearly documented. Revise the English.

**The procedure has been revised in Line 488-496. The details on the test procedure have been removed as advised by reviewer 2 (Line 497-513)**
Line 390-94. Why was the herd level variables first entered to MS access (unlike the animal level variables which are directly entered to MS excel) before being brought to MS excel for data cleaning and coding?

**Herd level variables were first entered into MS Access because of the large amount of data which needed to be compartmentalized in tables which could then be linked as appropriate (happens better in MS Access) before export to MS Excel for analysis as needed. Animal level variables were few and easy to enter into MS Excel in one sheet. Besides animal level data were in the sampling forms in the custody of the laboratory personnel while the herd level data were in questionnaires in the custody of data entry personnel in the epidemiology department. Entering the data separately saved time as one did not have to wait for the other. Linking the two data sets after entry was easy. See Line 524-525**

Line 402- To do this you need to document the se and sp of the test in the laboratory diagnosis section.

**Done in Line 492**
Line 403- 433 statistical analysis part has lot of repetition and unnecessary detail. For example line 405-6 has the same idea with line 402-405 both of them are about crude association using chi-square.

**Chi-square is used i) to test differences in proportions and ii) to test crude associations. The statement in Line 403-5 is about testing the difference in proportions and has been retained while that in Line405-6 is about testing the crude association between FMD sero-positivity and potential risk factors and has been removed in accordance with the general comments of the authors. The statistical analysis has been streamlined. See Line 539-541.**

But few lines down it mention bivariable analysis is done. The chi- square test of comparison and bivariable analysis are the same and the chi-square is not needed for variables in considered in the bivariable logistic regressions
**Removed as mentioned above**
The terminology bivariable is not correct has to be changed into univariable. The common usage is univariable and multivariable. It about the number of independent variables. If only one independent variable is included it is univariable and if more than one indnepenet variables is included it is multivariable.

**Corrected**
Line 434-36 The results of our study have been presented mainly in tables and figures and interpreted in text. Although the bivariable regression was carried out for all risk factors (individual animal and herd level) together, the results are in two tables to avoid too large a table.

**Sentences have been removed in Line 582-584**
Other issues in this section;
- The purpose the univariable analysis should be mentioned.

**Univariable analysis has been removed**
- For goodness of it test, it is enough to mention what tests are used with appropriate reference.

**Done in Line 571-573.** How each test measures goodness of fit is unnecessary detail.

**Unnecessary detail removed in Line 573-581**
- In several places in this part the word’ interpretation’ is incorrectly used. For example specifying confidence interval level and P- values (419-20) or putting results in text (line 434-35) are not interpretations. Interpretations ae given meaning for you findings that is done in the discussion.

**Terms have been removed in Line 565-6 and 582-3.**

**Results**

Line 450. In M&M, the number of herds sampled was stated as 323 herds* 2 strata = 646 so how the herd number increased to 898.

**If one herd could yield all the animals required, only that herd was sampled, otherwise additional herds that were in contact with the selected herds were sampled until the required number to be sampled in the sub-location was reached as also mentioned in Line 435-437. See also Line 602-603.**
Line 451. Mention why the remaining samples were not available for testing

**Mentioned in Line 604-605**

Line 472. However a large proportion ……

**Corrected on Line 626**

Line 482. Write in the full ideas instead of using “so” to make it clearer

**Corrected on Line 636 and 637**

Line 496. What is the difference between communal grazing and mixed grazing?

**Explained in Line 655**

Line 515-16. Sero-prevalence of a total of 7564 sera from the whole country was determined for the
presence of non-structural FMDV protein (antibodies).

**Corrected on Line 671-673**

Line 517. “Prevalence rate” Prevalence is not rate. Simply use prevalence (which known to be proportion). Correct this throughout the document.

**Corrected throughout the document**
Line 518. if χ2 has to be reported it has to include the degrees of freedom like χ2(3), which means χ2 at 3 degrees of freedom. Do this throughout the manuscript

**Where chi-square test is for comparison of two proportions at a time as in this case, the degree of freedom is 1 and is usually not reported. The results of chi-square test for test of crude association where the degrees of freedom are higher have been removed from the manuscript as per the reviewer recommendations.**
Line 516. Write it as apparent prevalence as you have also true prevalence estimate

**Corrected on Line 673**
Line 527. ‘Showed seronegativity’ is not good expression; state that prevalence in these counties is zero

**Corrected on Line 684-685**
Line 530. Put the estimated true Seroprevalence in figure.

**Inserted in Line 687**
Line 531-535. What does samplings sites represent, villages, individual herds or what? the map in figure 3 does not provide any information except the distribution of sampling sites. It should have indicate the negative sites as well. Moreover the legend and labels in the maps are not visible.

**Figure 3 has been removed. Explanation is now only in text in Line 689-691**

Line 539 PAIR WISE Pearson correlation…………

**Corrected on Line 785**

Line 539 …‘between county and production zone”….. No risk factor variable called ‘county type’ was mentioned in the list of variables indicated M&M part. **Included in L524**

Moreover test of collinearity is needed for the multivariable analysis. So this sentence should go down where multivariable results are documented.

**Inserted in Line 785-788**

Another issue here is the use of the term ‘risk factor” should be replaced by “potential risk factor”. The variables are hypothesized to be a risk factor, not yet confirmed to be risk factor. They will be risk factor when they are found so after the analysis. So they should be referred to as potential risk factors thought out the document until they are confirmed.

**Done throughout the document.**
Line 546-47. Where is the result of the statistical analysis i.e. P-value

**Provided on Line 705-6**

Line 546-53. In this section it seems χ2 is used but in chi-square for comparison of three or more groups, it tells you only whether there is significant difference in the proportion/ prevalence among all groups. It doesn’t tell you between which pairs is the difference significant. So how can you come up with this results where the variables has three categories like breed (exotic, local, cross)?

**The comparisons were of two categories at any time like local with cross-breed; local with exotic; cross-breed with exotic and not all three at the same time**.

Line 554. Replace ‘the between herd prevalence’ with ‘herd level prevalence”

**Done in Line 712**
Line 564. If it is not significant, why is it surprising? In the first place, in the result section just put what you find; such interpretation should be reserved for the discussion

**Corrected on Line 772**
Line 566-71. The same comment as above in line 546-53.

**Same explanation holds as for Line 546-53**
Line 604- 5. The result emphasis should be which factors are significantly associated with FMD seropositivty. Whether the association is positive or negative depends how the data was coded during analysis. For example, it is not sex that is negatively associated but it is being maleness.

**Corrected in Line 813-828**
Line 604. Instead of table 5, S4 and S5 should be part of the main manuscript not supplementary.

**All these tables have been removed from the manuscript.**
Line 612. S5 table attached is not about the herd level variables. Check whether the attached table is the right one.

**Table has been removed altogether**
Line 612-13. The comparator should be mentioned.

**The analysis has been removed**
Line 627-8. The purpose of univaraible analysis is to screen the potential risk factors. If cut off p-value 0.2 does not reduce them small cut off should have been used. Actually given the very large sample size there was no need to screen in this study and univaraible analysis was not necessary.

**Univariable analysis removed**
Line 645-76. Repetition of the table. Enough to mention the most salient results and leave the remaining detail to be referred from table

**Corrected**
**Discussion**
Line 685. Delete ‘It is true’ and start with ‘According to this study …..’

**Corrected on Line 870**
Line 724-30. For SR age of 6 months and above can be considered as matured as they can be bred at this age and classification of mature above 1years of age is not appropriate. Hence it difficult to think these age animals (age 6 months – 1 year) will be kept at homestead and are less exposed to the disease.

**Under Kenyan conditions, SR under one year are still not mature and are rarely bred. They remain close to the homestead or are grazed separate from the main herd.**

The observed higher prevalence in older animals could also be due cumulative seropositivity through repeated infection in their longer life time.

**Included in the discussion L917-918**
Line 730. Conversely

**Corrected on Line 920**
Line 731. ‘Female posted’ change the word ‘posted’ with other more appropriate word

**Corrected on Line 921**
Line 730-32. Both the univariable and the multivariable analysis showed females have significantly higher prevalence than males. Which result is being discussed here!
**Corrected on Line 920-923**
Line 740 ‘….position…’ to mean explanation?

**Removed**
Line 745. Change the word ‘contrary’ with the actual type purchase it self

**Removed**
Line 749-50. not clear

**Removed**
Line 755-59. Make it clearer

**Clarified on Line 945-952**
Line 768. … three..

**Removed**
Line 784-95. Merge this discussion with preceding paragraphs. In the first place, what should be discussed is the result of the final model where confounding is controlled. It is meaningless to discuss the crude results of the chi-square or univariable analysis as these can’t correctly identify significant risk factors because of confounding. So the discussion in the preceding paragraphs should be adjusted accordingly.

**Done**
Conclusion
Line 820-21. “The findings of this study give an understanding of the potential role of small ruminants in the epidemiology of FMD in Kenya and contribute to the global scenario’ Not exactly. It is difficult to say anything about the role of SR in epidemiology of FMD based on this study.

**Corrected in Line 1013-16**
Line 824-25. You found high Seroprevalence does not confirm that they transmit and maintain the disease. So this conclusion cannot be warranted based on your findings.

**Revised on line 1020**
Line 826. “Cross contamination” has to be replaced with cross transmission

**Replaced in Line 1021**
Line 827. ‘This outlays’?

**Corrected on Line 1022**
Line 829 ..”post”.. ??
**Corrected on Line 1024**
Figures
The legends and labels of all figures (fig.1,2,3) are not visible

**Fig. 1 and 3 have been removed and fig. 2 has been improved.**

Reviewer #2: This study aims to determine the seroprevalence of FMD in small ruminants and determine the risk factors associated. This is an original, highly useful study that may be utilised to further understand the epidemiology of FMD transmission in Kenya.
The manuscript is very thorough, however is much too long, particularly the introduction. The whole manuscript needs to be made more concise, as there is a lot of repetition throughout, between sections, between the text and figures, and between figures. Some tables could be merged, with some columns removed – for example tables 3 and S4. The current layout makes it more confusing to the reader than it needs to be – as this is a simple study approach with good results. However, the message seems to get lost in the length of the manuscript.
For the introduction, only information relevant to the study should be included, for example detail on molecular tests and differential diagnosis is not required – potentially only mentioned. Much of the information is or could be included in the discussion if it is necessary. Additionally, please ensure that you include references where appropriate, there are multiple sections where there are not enough references.
The methods section is very repetitive, particularly regarding the description of the number of counties, and the data analysis section could be much more concise. Additionally, the ELISA method does not need to be described in such detail as it is a commonly used test.
In the results section, please ensure that your subheadings accurately reflect what is included within them and avoid repetition between tables and the text. Additionally, are chi-squared test required if regression has been done? Or can this be more concisely displayed e.g. all in one table? At the moment it is very difficult to flip between all of the tables and the lengthy text to work out the results.
Additionally, I would like to see in the descriptive analysis the number of animals that were vaccinated for FMD and that had clinical signs (even if it was 0). This is very important information when investigating seroprevalence. Particularly when the vaccines administered in Kenya are often not of high quality and may induce NSP antibodies – this should also be mentioned in the discussion.
Additionally, please be careful with capitalised words that do not need to be, for example ‘county’ and directions (east/west etc.)
Consequently I recommend major revisions to this manuscript to make it more 'reader friendly'. **We appreciate the compliments regarding the study usefulness and thoroughness. The introduction has been shortened and irrelevancies removed. Repetitions have also been removed. S4 has been removed. Description of the ELISA method has been summarized. Chis-quare test of crude association results have been removed. In Kenya sheep and goats are not vaccinated against FMD as indicated in Line138 and 1015-16 No clinical signs were encountered during the survey as indicated now in Line 671. Unnecessary capitalization of words has been removed.**
