# Supplementary material for: Epidemiological study on foot-and-mouth disease in small ruminants: Sero-prevalence and risk factor assessment in Kenya
Source: PLoS One. 2021 Aug 2;16(8):e0234286. doi: 10.1371/journal.pone.0234286 (PMC8328338; doi:10.1371/journal.pone.0234286)
Supplement: Attachment — Submitted filename: Response to reviewer comments PONE-S-20-18913R1 SR FMD.docx [file pone.0234286.s006.docx]

We are sincerely grateful to both Reviewers for the valuable comments and suggetions that have helped us improve the manuscript and sharpen our writing skills. Below are our responses to the comments by the reviewers.

**Reviewer #1: Comments to authors

General comments.**

The manuscript has been considerably improved by the authors following the first round of comments. However, there are some issues remaining to be resolved. They are detailed below.
Note. The line numbers were not working in many cases. This made difficult to trace the corrections and has to be corrected in the next versions.

Thank you for the compliment. We apologize for the hardship faced in tracing corrections. In this revision, reference is made to the line numbers in the revised manuscript with track changes for ease of traceability. However the line numbers in the manuscript with accepted changes may be quite different because deleted lines in the revised manuscript with track changes remain while deleted lines in the manuscript with accepted changes disappear.
**Introduction**
Line 134. What have found to be the role of small ruminant in the epidemiology of FMD? Are they carrier transmitter, spill over hosts, primary host which can maintain the disease by themselves irrespective the presence of cattle or what? I don’t think the scope of the work can lead to these types of conclusions.

Statement has been removed in L139-143.
**Material and methods**
Line 184. A herd…..

Done on line 190.
Line 187. Instead of the software, provide reference for methodology of the sample size determination. Moreover, the website. http://www.promesa.co.nzl seems not working. check it.

Reference for formula used provided on Line 193.
Line 188. ‘expected between herd prevalence 30%” what does it mean and how is the 30% determined?

Corrected and reference provided on Line 196.
Line 190. Where did your get the intracluster correlation is 0.2?

Corrected and reference provided on Line 197.
Line 193. “Prevalence in a herd is 20%”. Give reference from where did you get this?

Reference provided on Line 203.
Generally, as which sample size formula is used is not mentioned, it is difficult to understand how sample size was determined.

Provided in Line 200-205.
Line 198. remove” Because it was one village per sub-location and one herd per village, it is appropriate to use the terms interchangeably” as it is confusing which terms it refers, and it also adds no clarity.

Removed in Line 209-210.
Line 224-227. multistage sampling; sublocation, village, herd and then individual animals. Compare this with your description in line 181-183.

Content of Line 224-227 has been removed in Line 236-238 to avoid confusion.
Line 308. (Pearson?) correlation coefficient of 0.3 threshold for collinearity is highly conservative. Are there any references for this?
Multicollinearity would have been more appropriate than simple collinearity.

It is acceptable to measure multicollinearity by the variance inflation factor (VIF) for continuous data or Spearman correlation for categorical data. Our data being mainly categorical could not utilize the VIF method. Reference [54] for correlation coefficient threshold is offered in Line 327.
**Results**
Line 334. Herd was defined better in M& M and not needed here.

Removed in Line 353.
Line 344. design effect for 2 may not be high for FMD as it id highly transmitted within a herd and could result high clustering effect.

Corrected to ‘sufficient’ on Line 611.
Line 403. “None of the herds surveyed had animals that showed FMD signs”. This should be qualified as at the time of sampling.
Done in Line 447-448.
Line 447. What are you doing in the above two tables? Were you not assessing association of risk factors for SR FMD? When you say FMD sero-positivity was higher in adult than young, don’t you mean age is a risk factor?! So the above analysis a univariable (crude) risk factor analysis and should come under risk factors analysis title. Actually, I don’t see its importance as far as you do the multivariable analysis as in table 5. After all the definitive test for risk factor is the multivariable test. This has been commented in the previous version.

The study is not just about association. It is important to know what the sero-positivities were across the categories with or without association and then find the association with the potential risk factors as the title indicates. It is acceptable to compare proportions without necessarily establishing association and strength of association with independent variables but in our study we have done both according to our objective.
Line 489. Table 5. This is the final model. Then why was the non-significant factors such as Brought in SR, SR breeding method, elevation included in the final model. In the methodology it was mentioned backward fitting (selection?) was used. If that is the case, in backward selection at each iteration the non-significant variables are removed until only significant factors and confounding factors remain in the final model.

The most parsimonious model together with interactions/contrasts has now been presented in Table 5.
Line 513. The interpretation for interaction is not clear. After the doing the interaction I would expect results such as: maleness and adultness interact positively and increased risk of FMD more than either of maleness or adultness alone.

This has been clarified in Line 585 to 591.
**Discussion**
Line 534. Across the categories.

Corrected in Line 676.
Line 583-684. Any evidence about subclinical infection SR to conclude this?

Do you mean Line 583-584?

Sentence has been removed in Line 683-687.
**Conclusion**
Line 600. What is this role?

Line 600 -604 your result could not give any clue about potential transmission of FMD between cattle and sheep let alone sheep as reservoir of infection for cattle. Is it not possible SR are getting infection from cattle? It is known that cattle are more susceptible to FMD and are often the maintenance hosts for FMD viruses except SAT viruses where African buffalo are involved. Cattle tend to be carrier longer and only short period of carrier was reported for SR.
This comment has been given in previous version as well but not has not been corrected. So should be corrected or need convincing explanation.

The content of these lines has been recast in the revised manuscript and unjustified assertions removed.

**Reviewer #2:** The authors have done a good job in working on the previous reviewer’s comments.

Thank you for the compliment.

Unfortunately, it seems some of my specific comments from review round 1 were not sent forward to the authors and therefore some that have not already addressed are repeated here again below.
Although the manuscript has been shortened, I still think there is a requirement to further make the introduction and results more concise, which will make the study more pleasurable to read, as the results are good. I think there are options for tables to be combined and the number of columns reduced so that the important results do not get lost (see specific comments below).
Additionally, from what I can see there is no description as to what S2 file 1 represents and therefore I think it should be removed.
**Specific comments
Abstract**
Line 31-32 – No need to capitalise ‘non-structural proteins’. And remove the second full stop after ‘kit’.

Done in Line 32-33.

Line 37 – Please provide the p number – not just say that it is was less than 0.05. Also, I think just the p value is enough, no need to include X2 value). However, I think there is no need to include this sentence (‘Sero-positivity was significantly……..p<0.05)’, as you have stated below that the sedentary production zone was negatively associated with seropositivity (line 40-41).

Sentence has been removed in Line 36-37.
**Introduction**
Needs shortening further and moving a few things around to make more concise. A focus on Kenya and small ruminants is recommended.
Line 72 – I do not think there is a need for Table 1. Or if so it should be included in the supplementary files. I think a short sentence about FMD prevalence on other East African countries would be enough here.

Table 1 taken out to be supplementary and sentence on prevalence of FMD in East African countries inserted in Line 71-75.
Lines 66-70 – Suggest removing this short paragraph, and instead mentioning seroprevalence studies of FMD in small ruminants in other East African countries.

Removed in Line 66-68 and summary sentence on sero-prevalence studies in East African countries inserted in Line 71-75.
Lines 75-108 – suggest reducing this further to make the introduction more concise. I think the most important thing to mention is FMD in small ruminants. (serotypes in Kenya are already mentioned below so no need to mention here?)

Removed in Line 89-92.
Lines 109-119 – Suggest removing this paragraph.

Removed in Line 115-126.
Lines 124 – 127 – Suggest removing these two sentences (‘Vaccination programmes…….maternal immunity’).

Removed in Line 130-133.
Lines 135 – 137 – suggest removing this sentence (‘This is because…..borders’).

Removed in Line 141-143.
Methods
Lines 151-158 – I think that this paragraph can be removed?

Removed in Line 157-164.
Line 161 – Add a full stop after ‘country’.

Done in Line 167.
Figure 1 – I’m not sure if it is just the version that is attached to this PDF, but please ensure Figure 1 is clear, as the version I can see is blurred.

I think it is the pdf version that is blurred.
Lines 183 – 184 – Suggest removing these sentences (‘The total sample……strata’). Also suggest using the term zone instead of strata going forward.

Done in Line 189-190. Zone applied instead of strata going forward.
Line 184 – please do not capitalise the word ‘herd’.

Done in Line 190.
Line 188-189 – please state why a 30% expected between herd prevalence was used, and in line 193, why an expected prevalence of FMD in the herd of 20% was used. What information was this based on?

Given in Line 196 and 203.
Lines 214-216 – suggest removing these two first sentences as this information has already been described previously.

Removed in Line 209-210.
Line 218 – change to ‘these WERE used…’

The ‘WAS’ is referring to the sampling frame which is one and not the sub-locations and clarified accordingly on Line 229.
Line 279 – 280 – Were two different ELISA tests performed or is the ‘3ABC blocking enzyme-linked immunosorbent assay’ the same as the ‘3 ABC- ELISA ID Screen®FMD NSP Competition kit’. I assume they are the same, but I think it is confusing here. Maybe it is best to say in line 275 that ‘Individual animal serum samples were analysed……’ and then remove the sentence in line 279-280 (‘At individual level……assay’).

Done in Line 287/88 and Line 291-293.
Line 287 – I do not think S2 file should be included – it is not very self-explanatory, and all of the data are in the other supplementary files.

File has been removed in Line 300.
Line 295 – What does this refer to? ‘--“Bunny-Wunnies Freak Out”’? Please remove.

Removed in Line 309.
Results
Line 340 – Please do not capitalise ‘counties’

Corrected in Line 359/360.
Lines 341-345 – Suggest moving this sentence and reasoning to the discussion.

Moved to line 607-612.
Suggest removing Table 2, as the important information is displayed in the other tables. Move to supplementary data if necessary to keep it. I am not sure what the column ‘sum’ refers to in Table 2. Is this table referring to the herd sizes that animals were sampled from, or the animals sampled?

The term ‘sum’ refers to number of sheep and goats in the herds sampled in the study area as indicated in the title but the term has been revised in the table and the title has been made clearer. The data in this table is not in any other table and therefore cannot be removed but is now placed as supplementary table 2.
Lines 355-398 – This section needs to be made more concise – suggest reducing to one paragraph. If theTable is brought to the main article instead of supplementary, only a short summary of results is required.

There are two Tables in this text which have now been brought to the main article (Table 1 and 2) and summaries given (Line 376-379 and Line 391-399). The tables cannot be combined because it would result in too large a table.
Lines 403-405 – Please improve the English language of this sentence.

Done in Line 447-449.
I’m not sure that confidence intervals are required for apparent sero-prevalence – only for true prevalence.

Because of the near perfection of the tests (Se=100%); Sp=99%). Calculation of the CIs of true prevalence$p\pm1.96\surd(\frac{\mathrm{pq}}{nJ^{2}})$ is equivalent to that of simple proportion p±1.96√pq/n as J (Se+Sp-1) nears 1. Thus CIs of true prevalence are similar to those of apparent prevalence and have been applied to the section in line 450-465.
Table 3 – Remove variable code. Suggest to remove the ‘negative’ column. Suggest adding p value column for each variable in Table 3 for chi squared test. Then the reader can clearly see the results without hunting through the text.

Table 4 – remove variable code. Suggest to remove the ‘negative’ column. Suggest adding p value column for each variable in Table 4 for chi-squared test. Then the reader can clearly see the results without hunting through the text.

In Table 3 and 4. Done for overall chi-square for each variable but for those with more than two categories, pairwise chi-square results have been given in the text as they cannot be included in the tables.
Can Tables 3 and 4 be combined?

No because combining them will create too large a table.
Lines 403-474 – Remove ?2 values from the text and only include p values. Also this section needs to be made more concise. Including the p values for the chi-squared tests in Tables 3 and 4 would mean less description is required. Done. However, for pairwise comparisons, chi-square remain in the text as they cannot be expressed in the Tables.

Line 479 – Do ‘all the potential risk factors’ mean all of those in Tables 3 and 4? Please be clear.

Clarified in Line 553.
Table 5 – Combine with Table 6? There are too many unnecessary columns. Suggest removing the columns B, Se/S.E. and Z ratio.

Table 5 combined with Table 6 and corrections made as suggested.
**Discussion**
Line 534 – ‘categories’ is spelled incorrectly.

Corrected in line 676.
Line 541 – change ‘FMD outbreak peaks is…’ to ‘FMD outbreaks are associated…’.

Corrected on Line 631.
Line 545 – Although foot and mouth disease is spread by contact, I would recommend changing this sentence as other methods of transmission are also common e.g. fomites/aerosol. It is more likely that as herd are closer together there are more chances for the virus to spread?

Corrected on Line 635-636.
Line 581 – ‘transmission’ is spelled incorrectly.

Corrected in Line 683.
Suggestion to include a discussion about the limitation of the study that testing was not done for circulating virus (e.g. virus isolation/PCR), and therefore it is unknown when these animals became infected. Indeed, further investigations (potentially using the same samples) could be done and also to identify the serotypes that the SR were infected with using serotype specific ELISAs.

Included in line 702-712.
Line 606 – Remove double word ‘cross’.

Removed in line 728.

7. PLOS authors have the option to publish the peer review history of their article ([what does this mean?](https://journals.plos.org/plosone/s/editorial-and-peer-review-process#loc-peer-review-history)). If published, this will include your full peer review and any attached files.


If you choose “no”, your identity will remain anonymous but your review may still be made public.


**Do you want your identity to be public for this peer review?** For information about this choice, including consent withdrawal, please see our [Privacy Policy](https://www.plos.org/privacy-policy).

Reviewer #1: No

Reviewer #2: No
